# Strong Electric Polarizability of Cone–Shell Quantum Structures for a Large Stark Shift, Tunable Long Exciton Lifetimes, and a Dot-to-Ring Transformation

**DOI:** 10.3390/nano13050857

**Published:** 2023-02-25

**Authors:** Christian Heyn, Leonardo Ranasinghe, Kristian Deneke, Ahmed Alshaikh, Carlos A. Duque, Wolfgang Hansen

**Affiliations:** 1 Center for Hybrid Nanostructures (CHyN), University of Hamburg, Luruper Chaussee 149, 22761 Hamburg, Germany; 2 Grupo de Materia Condensada-UdeA, Instituto de Física, Facultad de Ciencias Exactas y Naturales, Universidad de Antioquia UdeA, Calle 70 No. 52-21, Medellín AA 1226, Colombia

**Keywords:** droplet etching, quantum dot, quantum ring, photoluminescence, Stark shift, exciton lifetime, effective mass approximation

## Abstract

Strain-free GaAs cone–shell quantum structures (CSQS) with widely tunable wave functions (WF) are fabricated using local droplet etching (LDE) during molecular beam epitaxy (MBE). During MBE, Al droplets are deposited on an AlGaAs surface, which then drill low-density (about 1 × 10${}^{7}$ cm^−2^) nanoholes with adjustable shape and size. Subsequently, the holes are filled with GaAs to form CSQS, where the size can be adjusted by the amount of GaAs deposited for hole filling. An electric field is applied in growth direction to tune the WF in a CSQS. The resulting highly asymmetric exciton Stark shift is measured using micro-photoluminescence. Here, the unique shape of the CSQS allows a large charge–carrier separation and, thus, a strong Stark shift of up to more than 16 meV at a moderate field of 65 kV/cm. This corresponds to a very large polarizability of 8.6 × 10${}^{-6}$ eVkV ${}^{-2}$ cm^2^. In combination with simulations of the exciton energy, the Stark shift data allow the determination of the CSQS size and shape. Simulations of the exciton–recombination lifetime predict an elongation up to factor of 69 for the present CSQSs, tunable by the electric field. In addition, the simulations indicate the field-induced transformation of the hole WF from a disk into a quantum ring with a tunable radius from about 10 nm up to 22.5 nm.

## 1. Introduction

The electric-field induced polarization of charge carriers in semiconductor quantum structures is an important tool for the manipulation of the confined charge–carrier wave functions (WF). In optical systems with confined electrons and holes, the quantum-confined Stark effect allows for tuning the energy of the optical emission [1,2,3,4,5,6]. This enables, for instance, the resonant coupling of a semiconductor quantum dot (QD) into a Rb quantum memory [7,8]. For QDs that are symmetric in the electric-field direction, the Stark shift ΔE is often described by a power-law dependence [3,6]:

(1) $$\Delta E=E\left(F\right)-E\left(F=0\right)=-\mu F-\beta F^{2},$$

where μ is the dipole moment of the QD at zero field and β the polarizability. The origin of the Stark shift ΔE is a combination of a tilting of the energy bands and a spatial separation of the electron and hole wave functions (WF) in the electric field. For a general discussion, we take μ=0 and a linear dependence of the distance $d_{eh}$ between the WF barycenters in field direction and *F*. Then, a simple point–charge approximation yields $\Delta E=-d_{eh}eF=-\beta {\left(eF\right)}^{2}$ and, thus, $\beta =d_{eh}/\left(eF\right)$, with the elementary charge *e* [9]. This means that a strong Stark shift can be achieved either (i) by a high *F* or (ii) by a structure allowing a large $d_{eh}$. For (i), the maximum possible *F* is often limited by the onset of charge–carrier escape from the QDs [6]. This effect can be reduced by embedding the QDs in a matrix material, which provides a confining potential with higher energy barriers. Using this approach, a so-called giant Stark effect with ΔE up to 25 meV was demonstrated at a high *F* of 500 kV/cm [6]. For (ii), a large QD size can be used. However, this usually limits the possible quantization energies and, thus, the range of accessible emission wavelengths.

**Figure 1:**
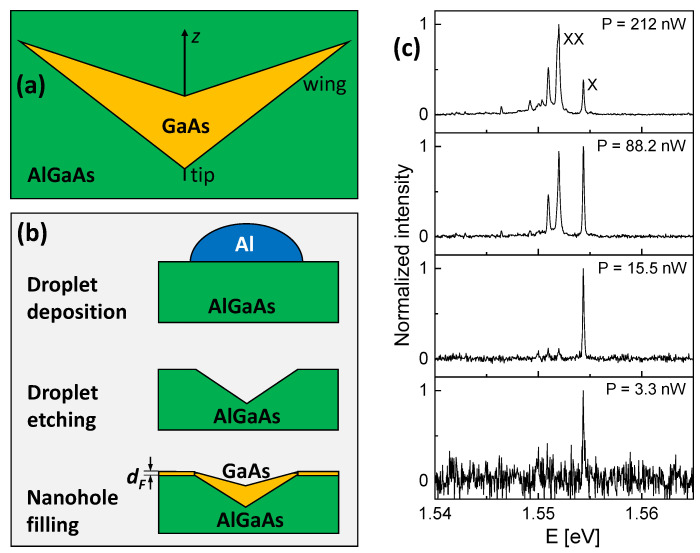
(**a**) Sketch of the cross section of a GaAs CSQS embedded in an AlGaAs matrix. The *z*-axis represents the growth direction and the direction of the applied electric field; (**b**) cross sectional sketch of the cone–shell quantum structure fabrication steps including Al droplet deposition, droplet etching, and filling of the nanoholes by deposition of a GaAs layer with thickness $d_{F}$; (**c**) PL spectra from a single GaAs CSQS with $d_{F}$ = 0.66 nm at varied laser power *P* as indicated. The exciton (X) and biexciton (XX) peaks are labeled. Here, the heterostructure is not covered with a top gate.

As an expedient, the cone–shell quantum structures (CSQS) studied here have a unique shape with a wing part allowing a large $d_{eh}$ and an indentation in the center, which reduces the effective structure size for high quantization energies. Figure 1a sketches the cross section of a CSQS. The electrons and holes are confined in the low band-gap GaAs region of the heterostructure. For the purposes of the discussion here, rotational symmetry around the growth direction (*z*-axis) is assumed, i.e., the 3D geometry of the CSQS forms a cone shell on which the electron and hole wave functions reside. The previous publications [10,11] describe the CSQS fabrication procedure and ref. [11] a first approach to determine the CSQS shape from atomic force microscopy (AFM) data. The present electric-field dependent data in combination with the simulation results allow a more precise CSQS shape determination and the evaluation of additional effects such as a controlled elongation of the radiative lifetime and a dot-to-ring transformation of the WFs. In CSQSs, the WFs are approximately localized at the surface of a cone. This unique shape can provide both a strong confinement for a high quantization energy as well as a large field-induced charge–carrier separation, e.g., for a strong Stark shift. As further exciting possibilities, the large charge–carrier separation in CSQSs exhibits a controlled elongation of the radiative lifetime and also the field-induced transformation from a disk-like into a ring-like WF with a tunable radius. We note that, due to the asymmetry in the growth direction, the CSQS have a finite zero-field polarization (μ>0). Moreover, the polarizability deviates from the simple parabolic Stark shift, which can, in a first approximation, be described by different polarizabilities β for the field ranges, where either the electrons or the holes reside in the wings of the CSQS.

In the next section, a brief description of the experimental setup including sample fabrication and the micro-photoluminescence (PL) setup is given. After that, the simulation model for the CSQS optical emission is described. This is followed by the results part with a discussion of the experimental and simulated Stark shift, the determination of the CSQS shape, and theoretical predictions of the field-controlled radiative lifetime and the field-induced quantum dot-to-ring transformation.

## 2. Experimental Setup

The fabrication of samples with a layer of GaAs CSQSs embedded in an AlGaAs matrix by local droplet etching (LDE) during molecular beam epitaxy (MBE) has been described in previous publications [10,11]. A schematic of the most relevant fabrication steps is shown in Figure 1b. In brief, a 50 nm thick Si-doped GaAs layer is deposited on a (001) GaAs substrate serving as back electrode for the electric-field dependent measurements. The next layer is a 120 nm thick AlGaAs barrier (the Al content is 33%). Now, the As flux is reduced, and cone-like nanoholes with a density of about $2\times 10^{7}$ cm^−2^ are drilled into the AlGaAs layer by self-assembled etching with Al droplets. The temperature during LDE is 618 °C and, for such process parameters, AFM inspection indicates an average depth of the nanoholes of $d_{H}$ = 24.5 nm and a radius of the nanohole opening of $r_{H}$ = 45 nm. The LDE nanoholes are used as a template for deposition of GaAs for CSQS generation, where the nominal thickness $d_{F}$ of the deposited GaAs filling layer defines the CSQS size. For the present samples, the value of $d_{F}$ is varied from 0.22–0.66 nm. We note that $d_{F}$ is not equal to the final height of the CSQS [10,11]. Finally, the CSQSs are capped by an 80 nm thick AlGaAs layer. After MBE growth, the top gates are realized by depositing a gate electrode consisting of 10 nm of Ti, 8 nm of Cr for better adhesion, and 30 nm of Au through physical vapour deposition on sample areas selected by photolithography.

A vertical electric field *F* is realized by a gate voltage $V_{g}$, which is applied between the integrated Si-doped GaAs back electrode and the metallic topgate. The distance between back electrode and topgate is $d_{g}$ = 200 nm. As explained in Section 4.1 in more detail, the applied gate voltage $V_{g}$ is converted in an electric field applied in the growth direction at the CSQS assuming a simple parallel–plate capacitor approximation.

For the micro-PL measurements, the samples are installed in an optical closed-cycle cryostat (Montana Cryostation S100) at a temperature of *T* = 4 K. For sample movement and CSQS selection, a stack of piezo motors is integrated inside the cryostat. The CSQS are optically excited by a green (532 nm) diode laser where the laser power *P* is adjustable by neutral density filters. The laser power is measured with a power meter, and the reading is corrected for the entrance window of the cryostat. The objective (Olympus LMPLFLN-BD, 100 × 0.8) for focusing the laser and collecting the light emitted from the sample is installed inside the cryostat. Due to the low QD density, individual QDs are selected without aperture by the focused laser. The QD emission is analyzed by a *f* = 500 mm monochromator in combination with an EMCCD camera for detection. Figure 1c shows examples of PL spectra from single CSQS at a varied excitation power *P*. The characteristic power dependence allows for identifying the exciton (X) and biexciton (XX) peaks according to [12].

## 3. Simulation Model

The GaAs CSQS are almost strain-free due to the negligible lattice mismatch between GaAs and the AlGaAs barrier material. This simplifies the modeling in comparison to strained InAs/GaAs quantum structures, where piezoelectric effects must be considered. In a previous publication [11], an advanced atomistic model [13,14] was used to study the influence of the size on the optical emission of CSQS. However, for the large number of simulation runs required in the present study, we switch here to a simpler but faster model [9,15] basing on effective mass approximation. The effective mass model assumes a rotational-symmetric quasi two-dimensional (2D) finite-element meshing [16] for the simulation of the electron and hole wave functions inside the CSQS and the corresponding eigenenergies, $E_{e}$ and $E_{h}$. The ultra-fine meshing and the computation of the energies and wave functions are carried out using the COMSOL-Multiphysics software. The potential is $V\left(r,z\right)=V_{0}\left(r,z\right)+\left(z-z_{0}\right)eF$ for electrons and $V\left(r,z\right)=V_{0}\left(r,z\right)-\left(z-z_{0}\right)eF$ for holes, where *r* is the radial position, *z* the position in direction perpendicular to the surface, $z_{0}$ the reference position for F=0, *F* is the electric field along *z*, and $V_{0}\left(r,z\right)$ the potential at zero field. This yields the single particle Hamiltonian $H_{e}=1/\left(2m_{e}^{*}\right){\left(i\hbar \vec{\nabla }\right)}^{2}+V_{0}\left(r,z\right)+\left(z-z_{0}\right)eF$ for electrons and $H_{h}=1/\left(2m_{h}^{*}\right){\left(i\hbar \vec{\nabla }\right)}^{2}+V_{0}\left(r,z\right)-\left(z-z_{0}\right)eF$ for holes, where $m_{e}^{*}$ is the electron effective mass, $m_{h}^{*}$ the hole effective mass, and ∇ the gradient operator. Without an electric field, the potential $V_{0}$ inside the GaAs CSQS is zero and inside the confining Al_0.33_Ga_0.67_As barrier $V_{0}$ = 0.286 eV for electrons and $V_{0}$ = 0.168 eV for holes (*T* = 4 K). The electron effective masses are $m_{e}^{*}=0.067m_{0}$ inside the GaAs CSQS and $m_{e}^{*}=0.086m_{0}$ inside the AlGaAs barrier, with the free electron mass $m_{0}$. For heavy holes, the masses are $m_{h}^{*}=0.51m_{0}$ and $m_{h}^{*}=0.59m_{0}$, respectively.

The 2D simulations provide cross sections through the rotational symmetric electron and hole wave functions. In the next step, the computed 2D wave functions are transformed into a three-dimensional (3D) cubic mesh for the calculation of the Coulomb interaction energy $C_{eh}$ via the Coulomb integral. Now, the energy of an exciton ground-state optical transition is taken from $E_{X}=E_{g}+E_{e}+E_{h}-C_{eh}$, with the GaAs band-gap energy $E_{g}$. We thus assume rotational symmetry of the charge density, which is expected to be a good approximation for small CSQDs. The effective mass approximation, on the other hand, becomes worse with decreasing QD size.

**Figure 2:**
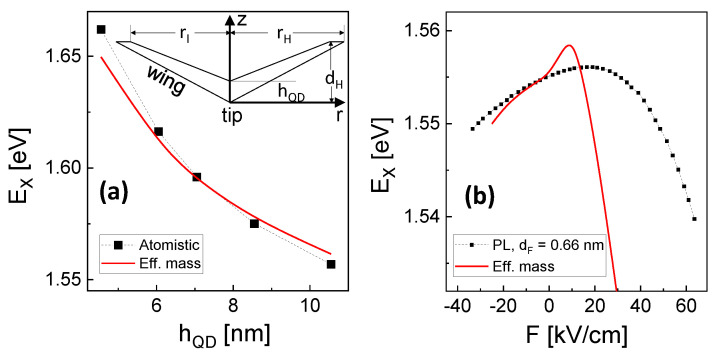
(**a**) Comparison of exciton energies $E_{X}$ as a function of the CSQS height $h_{QD}$ simulated with the atomistic model as reference (the data are taken from [11]) and with the present effective mass model. The inset shows a cross section of the assumed CSQS shape, with $d_{H}$ = 30 nm, $r_{H}$ = 56.5 nm, $r_{I}$ = 49.4 nm, and varied $h_{QD}$. For positive values of *F*, the electric field points from the tip to the base of the cone; (**b**) comparison of the measured exciton Stark shift of a sample with a deposited filling layer thickness $d_{F}$ = 0.66 nm with results of the effective mass model using the CSQS shape from (**a**) and $h_{QD}$ = 10.6 nm. This choice of $h_{QD}$ reproduces $E_{X}$ at *F* = 0.

To test the present model, Figure 2a compares simulated exciton energies with results from an atomistic model [11,13,14], which is considered as a reference. The data for the atomistic model are taken from [11]. Figure 2a demonstrates the good agreement between both models, which suggests the 2D effective mass model as a reasonable approximation for the present study. We note that the comparison assumes an approach for the CSQS shape [11], which is taken from atomic force microscopy (AFM) linescans (see the inset in Figure 2a). Since it turned out that this approach for the CSQS shape shows only a poor agreement with the experimental Stark shift data (see Figure 2b and Section 4.1), it will be modified in the following (see Section 4.2).

## 4. Results and Discussion

### 4.1. Stark Shift

**Figure 3:**
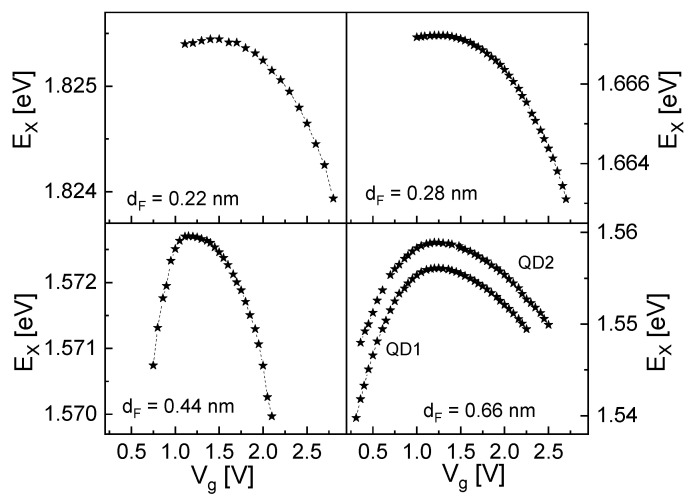
Measured exciton energies $E_{X}$ for CSQSs with varied filling layer thickness $d_{F}$ as a function of the gate voltage $V_{g}$. Data from two CSQSs (QD1, QD2) are shown for $d_{F}$ = 0.66 nm.

Figure 3 shows examples of measured exciton energies $E_{X}$ for CSQSs with four different $d_{F}$ as a function of the applied gate voltage $V_{g}$. Clearly visible is the the voltage dependent shift of the exciton energy, the Stark shift. With the increasing size of the CSQS in the field direction (increasing $d_{F}$), the exciton energy is reduced, and the slope of the Stark shift becomes stronger. Both effects agree with the behavior usually observed in semiconductor quantum structures, where a larger size reduces the quantization energy (and accordingly $E_{X}$) [11] and increases the Stark shift [17]. According to Equation (1) and the point–charge approximation, a larger CSQS (larger $d_{F}$) with a larger field-induced separation of the electron and hole wave functions allows a stronger Stark shift. The difference between QD1 and QD2 for $d_{F}$ = 0.66 nm in Figure 3 is probably caused by a slightly nonuniform shape and size distribution of the CSQSs due to the self-assembled fabrication process.

The conversion between $V_{g}$ and *F* is carried out by assuming a simple parallel plate capacitor as approximation: $F=-\left(V_{g}-V_{0}\right)/d_{g}$, with the distance $d_{g}$ = 200 nm between the integrated backgate and the metal topgate. The value $V_{0}$ reflects the built-in potential of the topgate Schottky contact and the zero-field polarization μ.

**Figure 4:**
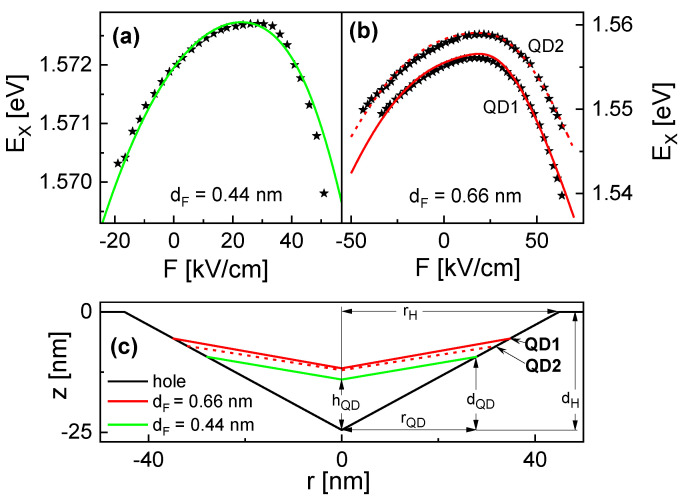
(**a**) Measured exciton energy $E_{X}$ (symbols) of a CSQS with $d_{F}$ = 0.44 nm as a function of *F* together with results of the effective mass simulation (line); (**b**) measured exciton energy $E_{X}$ (symbols) of two CSQS (QD1, QD2) with $d_{F}$ = 0.66 nm as a function of *F* together with simulation results (lines); (**c**) proposed cross-sectional shape of a nanohole and of the CSQSs from (**a**,**b**) determined by a comparison of measured and simulated Stark shift data. In the simulations, the reference position for F=0 is at $z_{0}=d_{H}/2$. The value of $d_{F}$ denotes the nominal layer thickness of the GaAs material, which is deposited for nanohole filling.

Since the contacts are not perfect due to interface charges, the built-in potential cannot be calculated easily from the material constants. Furthermore, the present CSQSs have a nonzero dipole moment and are asymmetric in field-direction, which further complicates the determination of $V_{0}$. Therefore, we use here a comparison with the simulation results to identify the value of $V_{0}$. For the samples shown in Figure 4, the such determined values of $V_{0}$ are 1.72 V ($d_{F}$ = 0.44 nm), 1.58 V ($d_{F}$ = 0.66 nm, QD1), and 1.63 V ($d_{F}$ = 0.66 nm, QD2). The slight differences are attributed to fluctuations of the topgate preparation procedure. Moreover, it considers a different zero-field polarization μ≠0 due to the dot asymmetry.

Figure 4a,b demonstrate the asymmetry of the Stark shift, where the highest $E_{X}$ is observed for F> 0, indicating a nonzero dipole moment and the curvature of $E_{X}\left(F\right)$ is stronger for positive *F* in comparison to negative fields. These effects are also visible in cone-shaped QDs [9] and attributed to the QD asymmetry in a growth direction. Electrons with smaller effective mass have a higher probability density also in the barrier material compared to the heavy holes. As a consequence, holes are more squeezed into the shape of the structure, such that the wave function barycenters are displaced already at zero *F*. A positive field $F_{0}$ compensates this effect, the distance between the wave function barycenters becomes zero, and ΔE=0. In addition, the different curvatures can be explained by the asymmetric dot shape and the different electron and hole effective masses. For $F>F_{0}$, holes are pushed into the wing part of the CSQS and electrons for $F<F_{0}$. Again, the respective effective mass controls that the hole wave function can be shifted more easily.

The largest measured Stark shift for the present samples is ΔE = 16.6 meV for $d_{F}$ = 0.66 nm (QD1 in Figure 4b), which is smaller than the so-called giant Stark effect with ΔE up to 25 meV in [6]. There, a high Stark shift is obtained by using an optimized barrier material which reduces the field-induced escape of charge carriers from a QD and allows very high fields up to 500 kV/cm. The reported value of β is 9.7 × 10${}^{-8}$ eV[kV]^−2^cm^2^ [6]. A fit of $E_{X}\left(F\right)$ for the present QD1 in Figure 4b with Equation (1) yields at F> 25 kV/cm a polarizability β = 8.6 ×$10^{-6}$ eV[kV]^−2^cm^2^, which is nearly two orders of magnitude higher. This suggests the CSQS as an alternative path towards a giant Stark effect but now at moderate fields by increasing the polarizability β.

**Figure 5:**
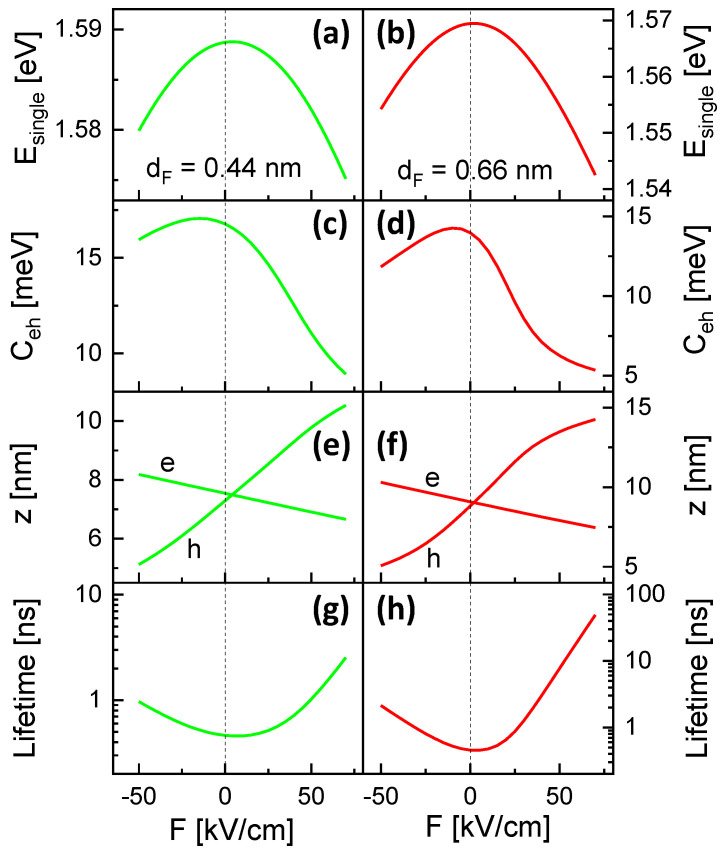
(**a**,**b**) Simulated single-particle energy $E_{single}$; (**c**,**d**) Coulomb interaction energy $C_{eh}$; (**e**,**f**) *z* positions of the electron (e) and hole (h) wave-function center of mass relative to the tip of the CSQS; and (**g**,**h**) radiative exciton lifetime $\tau_{X}$ for CSQSs with $d_{F}$ = 0.44 nm and 0.66 nm (QD1) as a function of *F*.

As a further result of this section, the PL data also demonstrate that the Stark shift of the biexciton peak is almost parallel to the exciton (the data are not shown here). This means that the splitting between the exciton and biexciton peaks [11] does not depend on *F*. In the single particle picture, the exciton energy is given by $E_{X}=E_{e}+E_{h}-C_{eh}$ and the biexciton energy by $E_{XX}=2E_{e}+2E_{h}-4C_{eh}+C_{ee}+C_{hh}$, with the repulsive electron–electron and hole–hole Coulomb interaction energies $C_{ee}$ and $C_{hh}$. The biexciton binding energy giving the splitting between the exciton and biexciton peaks is the energy gain when two excitons form a biexciton $2E_{X}-E_{XX}=2C_{eh}-C_{ee}-C_{hh}$. Thus, the observed field-independence of the splitting between the exciton and biexciton peaks indicates that the *F*-dependence of $C_{eh}$ (Figure 5c,d) is almost compensated by that of $C_{ee}$ and $C_{hh}$.

### 4.2. Shape of Cone–Shell Quantum Structures

The bottom part of the shape of a CSQS is given by the shape of the nanohole template, whereas the top part is controlled by capillarity during hole filling. In a previous publication [11], the CSQS shape was estimated from a series of AFM linescans of unfilled and filled nanoholes (inset in Figure 2a). However, since the AFM data are taken from different samples, this method is not very reliable in particular for the top part of the CSQSs. To test this shape, in Figure 2b, measured Stark shift data from QD1 in Figure 4b are compared with simulation results basing on the AFM data based shape. Obviously, the agreement is poor and the simulation predicts a too strong Stark shift. We attribute this discrepancy to the assumed CSQS shape approximation and correct the shape now by a comparison with the measured Stark shift data.

A starting point for the shape determination is the cone-like shape and size of the initial nanoholes, which is nicely reproducible even for different samples and was determined with a good statistics from a large number of AFM measurements: $d_{H}$ = 24.5 ± 1.8 nm and $r_{H}$ = 45 ± 3.7 nm. This defines the bottom part of the CSQS shape. For the top part, we have assumed in [11] that the GaAs which is filled into the nanohole completely covers the inner walls of the nanohole. This assumption yields a very pronounced wing part of the CSQS, as is illustrated in the inset of Figure 2a. This pronounced wing part allows a very strong charge carrier separation and, accordingly, predicts a very strong Stark shift as visible in the simulated behavior in Figure 2b. Since such a strong Stark shift is not observed experimentally, we assume now that there is no complete covering of the nanohole inner walls by the deposited GaAs and propose a new approximation for the CSQS shape.

The new shape approximation has three parameters: the CSQS radius $r_{QD}$, the height $h_{QD}$ at the center, and the outer height $d_{QD}$ (Figure 4c). Since the bottom part of a CSQS is formed by the initial nanohole, the ratio $d_{QD}/r_{QD}$ is assumed to be equal to $d_{H}/r_{H}$, which yields $d_{QD}=r_{QD}d_{H}/r_{H}≃0.54r_{QD}$. The remaining two parameters $r_{QD}$ and $h_{QD}$ are determined for the best agreement between the measured and simulated Stark shift.

Figure 4a,b demonstrate the very good reproduction of the experimental data for $d_{F}$ = 0.44 nm and $d_{F}$ = 0.66 nm by the simulation. The corresponding CSQS shapes are shown in Figure 4c, with $r_{QD}$ = 35.0 nm, $h_{QD}$ = 12.8 nm, and $d_{QD}$ = 19.1 nm for $d_{F}$ = 0.66 nm (QD1), $r_{QD}$ = 32.0 nm, $h_{QD}$ = 12.4 nm, and $d_{QD}$ = 17.4 nm for $d_{F}$ = 0.66 nm (QD2), and $r_{QD}$ = 28.0 nm, $h_{QD}$ = 10.45 nm, and $d_{QD}$ = 15.2 nm for $d_{F}$ = 0.44 nm. On the other side, the agreement for samples with smaller $d_{F}$ is not satisfactory. We assume that, for smaller structures, the shape deviates from the above approximation.

### 4.3. Field-Induced Charge–Carrier Separation

Figure 5a,b show the simulated single-particle energy $E_{single}=E_{g}+E_{e}+E_{h}$ and Figure 5c and d the Coulomb interaction energy $C_{eh}$. Both energies show a strong shift, where the shift $C_{eh}$ is caused by the field-induced separation of the electron and hole wave functions. For $E_{X}=E_{single}-C_{eh}$, the shift of $C_{eh}$ yields a flattening of the *F*-dependence as can be seen in Figure 3 and Figure 4.

Interestingly, the maximum of $E_{single}$ is observed at F>0 and that of $C_{eh}$ at F<0. The asymmetry of $E_{single}$ is related to a nonzero polarization at *F* = 0 due to the asymmetric shape of the CSQSs along the field direction and is discussed above in Section 4.1. The effect is illustrated by the simulated z-positions of the wave-function center of mass $z_{e}$ for electrons and $z_{h}$ for holes (Figure 5e,f). There, equal z positions for electrons and holes are found at a field F>0, which corresponds to the maximum of $E_{single}$. The reason for the significant asymmetry of $C_{eh}\left(F\right)$ is more complex. We assume that here the asymmetric shape of the CSQSs leads to asymmetric electron and hole WFs and a maximum $C_{eh}$ at $z_{e}\neq z_{h}$ (see Section 4.1). Furthermore, the simulations indicate an asymmetric *F*-dependent shift of $z_{e}$ and $z_{h}$, where $z_{e}\left(F\right)$ behaves almost linearly, and $z_{h}\left(F\right)$ shows a nonlinear and stronger displacement. For sphere, cone, and disk shaped QDs, previous simulations indicate an almost linear $d_{eh}\left(F\right)=\left|z_{e}\left(F\right)-z_{h}\left(F\right)\right|$ [9]. The nonlinear $z_{h}\left(F\right)$ of the present CSQSs can be explained by the transformation into a ring-like hole WF.

### 4.4. Radiative Lifetime and Quantum Ring Formation

A further effect of the strong field-induced separation of the electron and hole wave-functions is an elongation of the exciton lifetime $\tau_{X}$ up to radiative recombination. Assuming the strong-confinement regime and a homogeneous medium, lifetimes are calculated from the simulated WFs using [18] $\tau_{X}=3h^{2}c^{3}\epsilon_{0}m_{0}/\left(2n\pi e^{2}E_{X}^{2}f\right)$, with Planck’s constant *h*, the speed of light *c*, the vacuum permittivity $\epsilon_{0}$, the electron mass $m_{0}$, the refractive index *n* of the barrier material (AlGaAs), and the oscillator strength $f=E_{p}{\langle \psi_{e}|\psi_{h}\rangle }^{2}/E_{X}$. The latter is determined by the Kane energy $E_{p}$ and the overlap integral ${\langle \psi_{e}|\psi_{h}\rangle }^{2}$ between the electron and hole envelope wave functions in the CSQS. In this picture, the central quantity controlling $\tau_{X}$ is ${\langle \psi_{e}|\psi_{h}\rangle }^{2}$. Figure 5g,h show simulated lifetimes for CSQSs with $d_{F}$ = 0.44 nm and 0.66 nm. The data establish a strong elongation of $\tau_{X}$ with increasing |F|. Since Figure 5e,f indicate an almost symmetric charge–carrier separation $|z_{e}-z_{h}|$ around *F* = 0, the displacement along the *z*-direction cannot explain the significant asymmetry of $\tau_{X}$. We assume here an additional modification of ${\langle \psi_{e}|\psi_{h}\rangle }^{2}$ by the transition into a ring-like WF for the hole (see below). For QD1 in Figure 4b, optical emission is detected up to *F* = 65 kV/cm. The predicted lifetime at this field is $\tau_{X}$ = 31 ns, which is 69 times longer compared to the shortest lifetime $\tau_{X}$ = 0.453 ns at *F* = 5 kV/cm.

**Figure 6:**
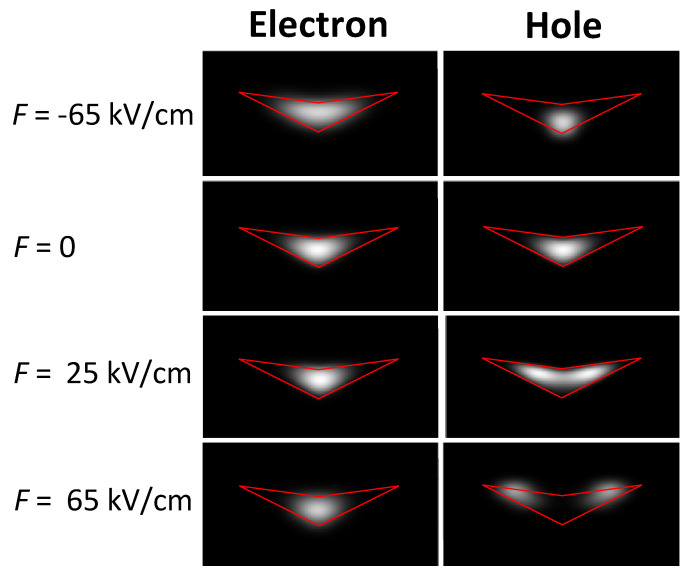
Grey-scale plots of cross sections through the simulated electron and hole probability densities $\psi_{e}^{2}$, $\psi_{h}^{2}$ inside of a CSQS (QD1, red lines) at different vertical electric fields *F*.

Simulated electron and hole probability densities $\psi_{e}^{2}$, $\psi_{h}^{2}$ inside of a CSQS are shown in Figure 6 for different values of *F*. At *F* = 0, $\psi_{e}^{2}$ and $\psi_{h}^{2}$ are approximately shaped like a disk. For *F* = −65 kV/cm, $\psi_{e}^{2}$ is only slightly changed, whereas $\psi_{h}^{2}$ changes into a sphere-like shape with barycenter shifted to the tip of the CSQS. More interesting is the behavior at *F* = 65 kV/cm. Here, the hole $\psi_{h}^{2}$ is transformed into a ring shape with a radius of approximately 20.5 nm. The radius can be tuned by the applied field, where a ring shaped $\psi_{h}^{2}$ is observed for F≥ 25 kV/cm with a radius of about 10 nm up to the above 20.5 nm at *F* = 65 kV/cm. In particular, this controlled transformation of an isolated charge carrier from a disk into a ring shape with tunable radius by a vertical electric field is an intriguing feature of the present CSQSs.

## 5. Conclusions

The studied cone–shell quantum structures exhibit a strong electric polarizability, which allows a wide tunability of the electron and hole wave functions. This has several unique and intriguing consequences. A strong Stark shift of the exciton and biexciton peaks up to more than 16 meV at a field of 65 kV/cm allows for tailoring the emission energy for instance for a resonant coupling in a Rb quantum memory [7,8]. The large carrier separation represents an alternative strategy to achieve a strong Stark effect at moderate fields. The elongated lifetimes can be interesting for a “PL on demand”, where we expect even longer lifetimes for optimized structures. We propose a concept for applications, where after loading a CSQS with an exciton at F≫ 0, the instant of the optical emission is controlled by switching *F* to zero. Finally, semiconductor quantum rings represent a fascinating class of quantum structures with interesting properties [19]. A prominent example is quantum–interference phenomena in the rings, the so-called Aharonov–Bohm effect [20], which have attracted a lot of interest. A such induced oscillatory persistent current carried by a single electron in a quantum ring was measured by means of ultrasensitive magnetization experiments [21]. Optically active quantum rings are usually geometry defined and often created using advanced self-assembly methods [22,23,24]. Obviously, the diameter of the ring there is almost fixed. As a novel aspect in this field, we predict that for the present CSQSs the hole wave function can be transformed by a field from a disk to a ring with a tunable diameter.

## Data Availability

The data presented in this study are available on request from the corresponding author.

## References

[1] Mendez E., Bastard G., Chang L., Esaki L., Morkoc H., Fischer R. (1982). Effect of an electric field on the luminescence of GaAs quantum wells. Phys. Rev..

[2] Miller D.A.B., Chemla D.S., Damen T.C., Gossard A.C., Wiegmann W., Wood T.H., Burrus C.A. (1984). Band-Edge Electroabsorption in Quantum Well Structures: The Quantum-Confined Stark Effect. Phys. Rev. Lett..

[3] Empedocles S.A., Bawendi M.G. (1997). Quantum-Confined Stark Effect in Single CdSe Nanocrystallite Quantum Dots. Science.

[4] Heller W., Bockelmann U., Abstreiter G. (1998). Electric-field effects on excitons in quantum dots. Phys. Rev..

[5] Finley J.J., Sabathil M., Vogl P., Abstreiter G., Oulton R., Tartakovskii A.I., Mowbray D.J., Skolnick M.S., Liew S.L., Cullis A.G. (2004). Quantum-confined Stark shifts of charged exciton complexes in quantum dots. Phys. Rev..

[6] Bennett A.J., Patel R.B., Joanna S.S., Christine A.N., David A.F., Andrew J.S. (2010). Giant Stark effect in the emission of single semiconductor quantum dots. Appl. Phys. Lett..

[7] Akopian N., Wang L., Rastelli A., Schmidt O.G., Zwiller V. (2011). Hybrid semiconductor-atomic interface: Slowing down single photons from a quantum dot. Nat. Photonics.

[8] Keil R., Zopf M., Chen Y., Höfer B., Zhang J., Ding F., Schmidt O.G. (2017). Solid-state ensemble of highly entangled photon sources at rubidium atomic transitions. Nat. Commun..

[9] Heyn C., Ranasinghe L., Zocher M., Hansen W. (2020). Shape-Dependent Stark Shift and Emission-Line Broadening of Quantum Dots and Rings. J. Phys. Chem..

[10] Heyn C., Stemmann A., Köppen T., Strelow C., Kipp T., Grave M., Mendach S., Hansen W. (2009). Highly uniform and strain-free GaAs quantum dots fabricated by filling of self-assembled nanoholes. Appl. Phys. Lett..

[11] Heyn C., Gräfenstein A., Pirard G., Ranasinghe L., Deneke K., Alshaikh A., Bester G., Hansen W. (2022). Dot-Size Dependent Excitons in Droplet-Etched Cone-Shell GaAs Quantum Dots. Nanomaterials.

[12] Graf A., Sonnenberg D., Paulava V., Schliwa A., Heyn C., Hansen W. (2014). Excitonic states in GaAs quantum dots fabricated by local droplet etching. Phys. Rev..

[13] Bester G., Nair S., Zunger A. (2003). Pseudopotential calculation of the excitonic fine structure of million-atom self-assembled InGaAs/GaAs quantum dots. Phys. Rev..

[14] Bester G. (2008). Electronic excitations in nanostructures: An empirical pseudopotential based approach. J. Phys. Condens. Matter.

[15] Heyn C., Küster A., Ungeheuer A., Gräfenstein A., Hansen W. (2017). Excited-state indirect excitons in GaAs quantum dot molecules. Phys. Rev..

[16] Melnik R.V.N., Willatzen M. (2004). Bandstructures of conical quantum dots with wetting layers. Nanotechnology.

[17] Vina L., Mendez E.E., Wang W.I., Chang L.L., Esaki L. (1987). Stark shifts in GaAs/GaAlAs quantum wells studied by photoluminescence spectroscopy. J. Phys. Solid State Phys..

[18] Tighineanu P., Daveau R., Lee E.H., Song J.D., Stobbe S., Lodahl P. (2013). Decay dynamics and exciton localization in large GaAs quantum dots grown by droplet epitaxy. Phys. Rev..

[19] Fomin V.M. (2018). Physics of Quantum Rings.

[20] Aharonov Y., Bohm D. (1959). Significance of Electromagnetic Potentials in the Quantum Theory. Phys. Rev..

[21] Kleemans N.A.J.M., Bominaar-Silkens I.M.A., Fomin V.M., Gladilin V.N., Granados D., Taboada A.G., García J.M., Offermans P., Zeitler U., Christianen P.C.M. (2007). Oscillatory Persistent Currents in Self-Assembled Quantum Rings. Phys. Rev. Lett..

[22] Garcia J.M., Medeiros-Ribeiro G., Schmidt K., Ngo T., Feng J.L., Lorke A., Kotthaus J., Petroff P.M. (1997). Intermixing and shape changes during the formation of InAs self-assembled quantum dots. Appl. Phys. Lett..

[23] Stemmann A., Koeppen T., Grave M., Wildfang S., Mendach S., Hansen W., Heyn C. (2009). Local etching of nanoholes and quantum rings with InxGa1-x droplets. J. Appl. Phys..

[24] Llorens J.M., Wewior L., Cardozo de Oliveira E.R., Ulloa J.M., Utrilla A.D., Guzmán A., Hierro A., Alén B. (2015). Type II InAs/GaAsSb quantum dots: Highly tunable exciton geometry and topology. Appl. Phys. Lett..

